# Identification and functional expression of the pepper RING type E3 ligase, CaDTR1, involved in drought stress tolerance via ABA-mediated signalling

**DOI:** 10.1038/srep30097

**Published:** 2016-07-21

**Authors:** Hyunhee Joo, Chae Woo Lim, Sung Chul Lee

**Affiliations:** 1 Department of Life Science (BK21 program), Chung-Ang University, 84 Heukseok-Ro, Dongjak-Gu, Seoul 156-756, Republic of Korea

## Abstract

Drought negatively affects plant growth and development, thereby leading to loss of crop productivity. Several plant E3 ubiquitin ligases act as positive or negative regulators of abscisic acid (ABA) and thus play important roles in the drought stress response. Here, we show that the C3HC4-type RING finger E3 ligase, CaDTR1, regulates the drought stress response via ABA-mediated signalling. CaDTR1 contains an amino-terminal RING finger motif and two carboxyl-terminal hydrophobic regions; the RING finger motif functions during attachment of ubiquitins to the target proteins, and the carboxyl-terminal hydrophobic regions function during subcellular localisation. The expression of *CaDTR1* was induced by ABA, drought, and NaCl treatments. CaDTR1 localised in the nucleus and displayed *in vitro* E3 ubiquitin ligase activity. *CaDTR1*-silenced pepper plants exhibited a drought-sensitive phenotype characterised by high levels of transpirational water loss. On the other hand, *CaDTR1*-overexpressing (OX) Arabidopsis plants exhibited an ABA-hypersensitive phenotype during the germinative and post-germinative growth stages. Moreover, in contrast to *CaDTR1*-silenced pepper plants, *CaDTR1*-OX plants exhibited a drought-tolerant phenotype characterised by low levels of transpirational water loss via increased stomatal closure and high leaf temperatures. Our data indicate that CaDTR1 functions as a positive regulator of the drought stress response via ABA-mediated signalling.

Plants are frequently challenged by environmental stresses, which inhibit plant growth and development. Among these adverse environmental cues, drought stress presents a serious threat to plant survival. Consequently, plants have established elaborate defence mechanisms to enable survival and adaptation under water-deficit conditions^1^. Under drought-stress conditions, plants maximise water retention by minimising transpiration from the leaves and maximising water uptake from the roots^2^,^3^. The physiological and molecular strategies underlying drought stress have been extensively investigated; however, plant defence mechanisms constitute a complex phenomenon, and the precise functional modifications induced by drought stress remain unclear. When plants perceive a drought signal through sensors, they trigger expression of stress-related genes and accumulation of the plant hormone abscisic acid (ABA)^1^,^4^,^5^. ABA plays a key role in response to biotic and abiotic stresses, by inducing many molecular alterations^5^,^6^. Moreover, ABA regulates expression of numerous stress-related genes and synthesis of diverse proteins—including transcription factors and E3 ligases—to enable survival via changes in stomatal aperture, osmotic adjustment, and modifications of root hydraulic conductivity^7^,^8^. In comparison with other plant defence hormones, ABA regulates a large number of genes; more than 10% of Arabidopsis genes are controlled by ABA^9^,^10^. Several genetic studies using ABA-sensitive or ABA-insensitive mutants have identified various ABA-signalling components from perception to response^11^,^12^,^13^,^14^.

Protein degradation via ubiquitination is an important post-translational modification in eukaryotes^15^,^16^,^17^. In plant cells, the ubiquitin–26S proteasome pathway is involved in mediation of various hormone signals, including perception of auxin, gibberellins and jasmonate, and transduction in the ethylene- and ABA-signalling pathways^18^,^19^,^20^,^21^. Ubiquitination is a multi-step process for covalent attachment of ubiquitin to the target protein, and it operates through the sequential action of three enzymes—E1 (ubiquitin-activating enzyme), E2 (ubiquitin-conjugating enzyme), and E3 (ubiquitin ligase). The key factor for determining substrate specificity is E3, which interacts with and transfers ubiquitin from E2 to the target protein^17^,^22^,^23^,^24^. The Arabidopsis genome encodes more than 1,400 different E3 ubiquitin ligases, including more than 470 Really Interesting New Gene (RING) domain-containing E3 ubiquitin ligases^23^,^25^. An increasing number of studies have indicated that protein degradation via RING type E3 ligases plays a critical role in ABA signalling^26^,^27^. For example, AIP2 and KEEP ON GOING function as negative regulators of ABA by mediating degradation of transcription factor ABI3 and ABI5, respectively^26^,^28^,^29^. In contrast, SDIR1 functions as a positive regulator of ABA by promoting SDIRIP1 degradation^30^,^31^.

In the present study, we isolated and characterised the E3 ubiquitin ligase gene, *CaDTR1* (*Capsicum annuum* Drought Tolerance RING 1), which contains a RING finger motif and is induced in pepper leaves in response to ABA, dehydration, and high-salinity treatments. The CaDTR1 protein localised in the nucleus and displayed *in vitro* E3 ubiquitin ligase activity. Based on the expression patterns of *CaDTR1*, we used virus-induced gene silencing (VIGS) and overexpression of *CaDTR1* in pepper and Arabidopsis, respectively, to elucidate the functions of CaDTR1 in response to drought stress. We found that *CaDTR1*-silenced pepper plants exhibited an ABA-insensitive and drought-sensitive phenotype characterised by high levels of transpirational water loss. In contrast, *CaDTR1*-overexpressing (OX) Arabidopsis plants exhibited an ABA-hypersensitive and drought-tolerant phenotype. Our data indicate that CaDTR1 functions as a positive regulator of the drought stress response via ABA-mediated signalling.

## Results

### Identification of the CaDTR1 protein as an E3 ubiquitin ligase

We used RNA-seq analysis to isolate *CaDTR1* (accession no. KU557245) from the leaves of pepper plants that had been subjected to drought stress treatment. The putative *CaDTR1* cDNA consists of a 660-bp open reading frame, and it encodes 219 amino acid residues with a calculated molecular mass of 23.7 kD and an isoelectric point (pI) of 6.57. The putative protein encoded by *CaDTR1* contains a highly conserved C3HC4 type RING finger motif (residues 26–67) in the N-terminal region and two predicted membrane domains (residues 137–159 and 201–218) in the C-terminal region (C-term). The RING finger motif is essential for E3 ligase activity in the ubiquitin–26S proteasome system. The results of multiple sequence alignment revealed that CaDTR1 has relatively high amino acid sequence identity (57–92%) with other RING finger proteins (Supplementary Fig. 1).

**Figure 1:**
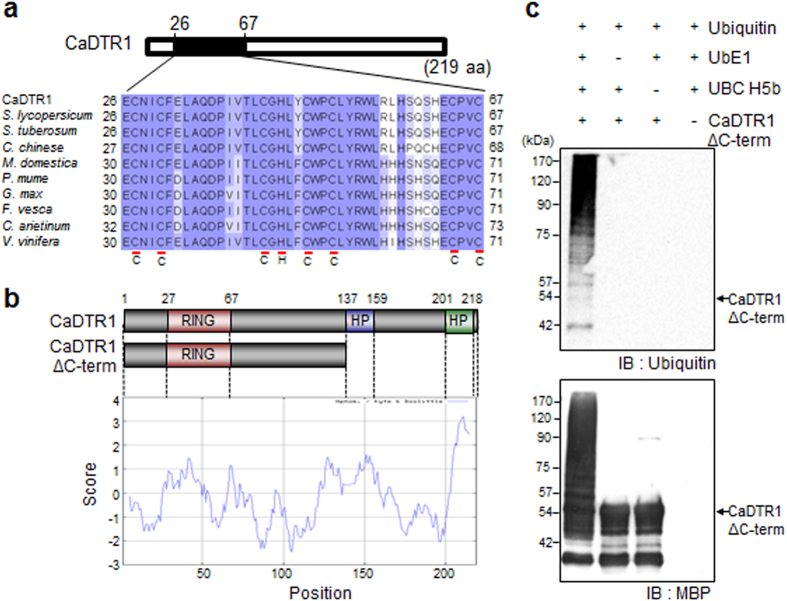
Amino acid sequence analysis and *in vitro* auto-ubiquitination of pepper CaDTR1 (*Capsicum annuum* Drought-Tolerant RING finger protein 1). (**a**) Alignment of the RING zinc finger C3HC4-type domain. Conserved cysteine (C) and histidine (H) residues are indicated using underlines. (**b**) Schematic representation of the CaDTR1 proteins with or without hydrophobic regions (HP), and hydrophobicity index. (**c**) Auto-ubiquitination of CaDTR1. In the presence of ubiquitin, E1 (UbE1), and E2 (UBCH5b), maltose-binding protein (MBP)–CaDTR1 fusion proteins displayed E3 ubiquitin ligase activity. Detection of MBP–CaDTR1 auto-ubiquitination. MBP–CaDTR1 fusion proteins were detected using ubiquitin and MBP antibodies; shifted bands indicate the attachment of ubiquitin molecules.

Several RING finger proteins are known to display *in vitro* E3 ligase activity^14^,^30^,^32^. CaDTR1 contains a RING finger motif (Fig. 1b), and therefore we performed an *in vitro* self-ubiquitination assay to determine whether CaDTR1 acts as an E3 ligase (Fig. 1c). We expressed the CaDTR1ΔC-term protein in *E. coli* as a fusion protein with maltose-binding protein (MBP), and we subsequently used affinity chromatography to purify CaDTR1ΔC-term-MBP from the soluble fraction of total proteins. We used human E1 and E2 for the *in vitro* ubiquitin ligase activity assay. The ubiquitinated proteins were detected using anti-ubiquitin and anti-MBP antibodies. We found that CaDTR1ΔC-term–MBP displayed E3 ubiquitin ligase activity in the presence of E1 and E2, indicating that CaDTR1 functions as an E3 ubiquitin ligase.

### Induction of *CaDTR1* expression in pepper leaves in response to abiotic stresses

**Figure 2:**
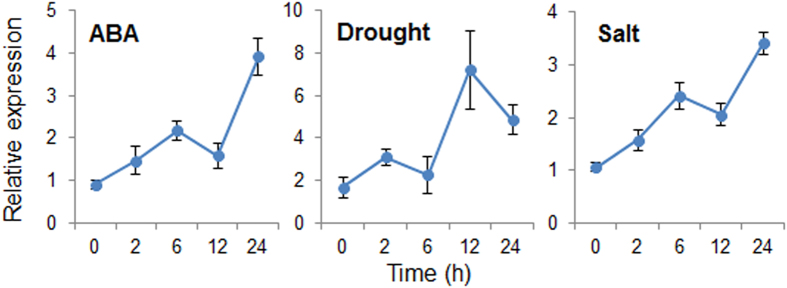
Expression of the *CaDTR1* gene. The expression pattern of the *CaDTR1* gene was analysed in the leaves of pepper plants after treatment with 100 μM abscisic acid (ABA), drought, or 200 mM NaCl. The pepper *Actin1* gene was used as an internal control.

To monitor the expression of *CaDTR1* under abiotic stress conditions, we performed qRT-PCR analysis using pepper leaves that had been subjected to ABA, dehydration, and high-salinity treatments (Fig. 2). First, we investigated the transcript levels of *CaDTR1* after ABA treatment. We found that the *CaDTR1* transcripts were weakly expressed after 2 h of ABA treatment and reached their maximum levels after 24 h. ABA is known to play a key role in the abiotic stress response; moreover, ABA and abiotic stress signals share common components in their signal transduction pathways^33^. Hence, we investigated the expression levels of *CaDTR1* transcripts in pepper leaves that had been subjected to dehydration and NaCl treatments. The results of qRT-PCR analysis revealed the upregulation of *CaDTR1* in pepper leaves that had been subjected to dehydration treatment. In addition, we found that the steady-state levels of *CaDTR1* transcripts were slightly upregulated after NaCl treatment.

### Localisation of the CaDTR1 protein in the nucleus

**Figure 3:**
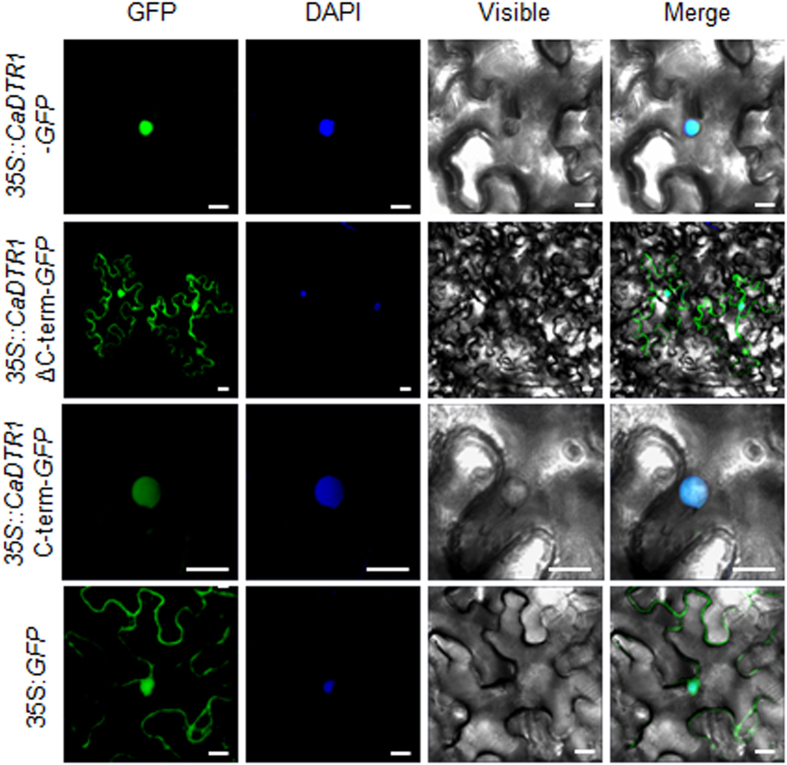
Subcellular localisation of CaDTR1 based on transient expression of the green fluorescent protein (GFP) fusion protein in *Nicotiana benthamiana* epidermal cells. The 35S:*CaDTR1*-*GFP*, 35S:*CaDTR1*ΔC-term-*GFP* (amino acids 1–136), 35S:*CaDTR1*C-term-*GFP* (amino acids 137–219) and 35S:*GFP* constructs were expressed using agroinfiltration of *N. benthamiana* leaves and was observed under a confocal laser-scanning microscope. DAPI staining was used as a marker for the nucleus. White bar = 20 μm.

Several E3 ubiquitin ligases are known to function in the cytoplasm and nucleus^24^,^30^. To determine the subcellular localisation of the CaDTR1 protein in plant cells, we generated fusion proteins between CaDTR1 and the green fluorescent protein (GFP) gene under the control of the 35S promoter. Transient expression of the *35S:CaDTR1*-*GFP* construct showed that the CaDTR1 protein was localised in the nucleus of *Nicotiana benthamiana* epidermal cells (Fig. 3). CaDTR1 contains two TM domains (amino acids 137–159 and 201–218) in the C-terminal region. To verify whether C-terminal region function in the subcellular localisation of CaDTR1, we performed deletion analysis using the *35S:CaDTR1ΔC-term-GFP* and *35S:CaDTR1C-term-GFP* constructs. The fluorescent signals of *CaDTR1ΔC-term* were detected in the nucleus and cytoplasm, and *CaDTR1C-term-GFP* fusion protein was localised in the nucleus, indicating that the C-terminal region is essential for subcellular localisation of the CaDTR1 protein in the nucleus.

### Increased drought sensitivity of *CaDTR1*-silenced pepper plants

**Figure 4:**
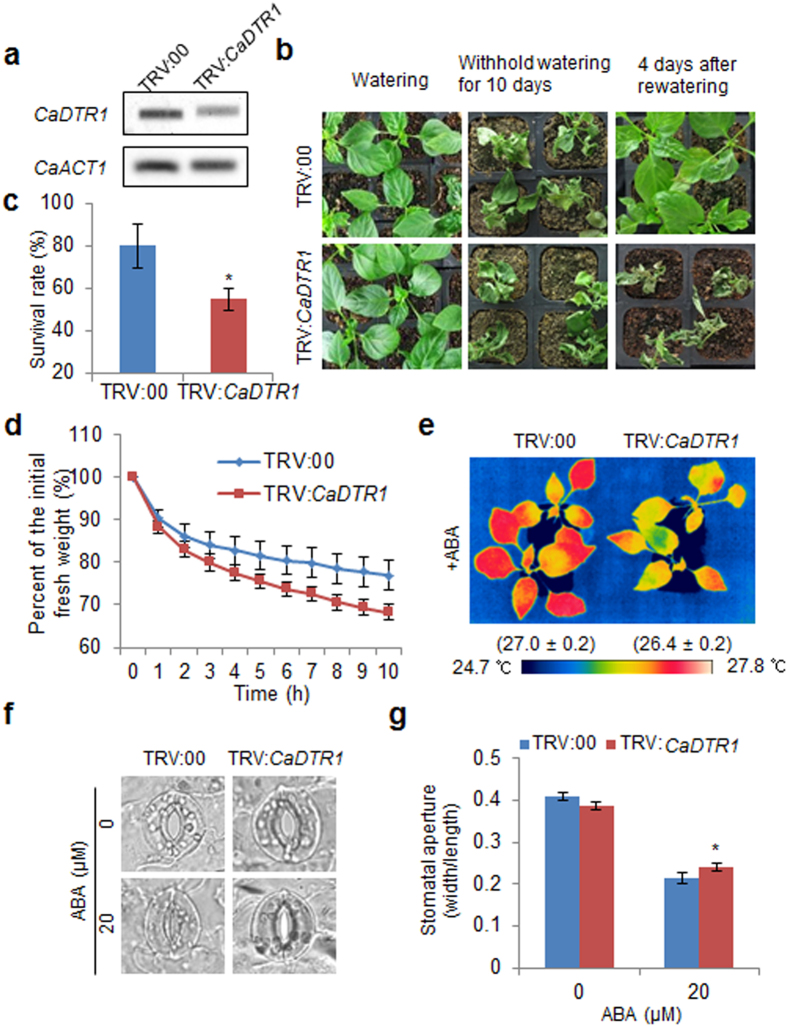
Reduced drought tolerance of *CaDTR1*-silenced pepper plants. (**a**) RT-PCR analysis of *CaDTR1* expression in the leaves of pepper plants transfected with the empty vector control (TRV:00) and *CaDTR1*-silenced constructs (TRV:*CaDTR1*). *CaACT1* was used as an internal control gene. (**b**) The drought-sensitive phenotype of *CaDTR1*-silenced pepper plants. Control and *CaDTR1*-silenced pepper plants were grown in pots for 6 weeks under favourable conditions. Thereafter, watering was withheld for 10 days, followed by re-watering for 4 days. (**c**) Survival rates of control and *CaDTR1*-silenced pepper plants after 4 days of re-watering. Data represent the mean ± standard error of three independent experiments, each evaluating 20 plants. (**d**) Transpirational water loss from the leaves of empty vector control and *CaDTR1*-silenced pepper plants at various times after detachment of leaves. (**e**) Decreased leaf temperatures of *CaDTR1*-silenced pepper plants in response to ABA treatment. (**f**,**g**) Stomatal apertures in control and *CaDTR1*-silenced pepper plants treated with ABA. Leaf peels were harvested from 3-week-old plants of each line and incubated in stomatal opening solution (SOS) buffer containing 0 μM and 20 μM ABA. Representative images were taken under a microscope and the stomatal apertures were measured. Data represent the mean ± standard error of three independent experiments. Asterisks indicate significant differences between three independent experiments (Student’s *t*-test; P < 0.05).

Abiotic stress treatments induced the expression of *CaDTR1* transcripts in the leaves of pepper plants (Fig. 2), and therefore we performed virus-induced gene silencing (VIGS)-based gene function analysis to determine the *in vivo* function of CaDTR1. In comparison with empty vector control plants (TRV:00), *CaDTR1*-silenced pepper plants (TRV:*CaDTR1*) accumulated low levels of *CaDTR1* transcripts (Fig. 4a). Next, we investigated the function of CaDTR1 in response to drought stress. Under well-watered and drought-stress conditions, we observed no phenotypic differences between control plants and *CaDTR1*-silenced pepper plants (Fig. 4b, left and middle panel). However, upon re-watering, the *CaDTR1*-silenced pepper plants exhibited a more wilted phenotype than the control plants (Fig. 4b, right panel); hence, only 55% of the *CaDTR1*-silenced plants survived, but 80% of the control plants resumed their growth (Fig. 4c). To determine whether transpirational water loss affects the drought-sensitive phenotype of *CaDTR1*-silenced pepper plants, we measured the leaf fresh weight of detached pepper leaves (Fig. 4d). At 10 h after detachment, the leaf fresh weight was significantly lower in *CaDTR1*-silenced pepper plants (68%) than in control plants (77%). Previous studies have suggested that drought tolerance is related to ABA sensitivity^14^,^34^,^35^,^36^; therefore, we examined the ABA responses of *CaDTR1*-silenced pepper plants (Fig. 4e–g). First, we monitored the leaf temperatures of pepper plants after treatment with 50 μM ABA. The leaf temperatures of *CaDTR1*-silenced pepper plants were lower than those of control plants (Fig. 4e). Stomatal opening and closure leads to an increase and decrease in evaporative cooling, respectively, thereby affecting the leaf temperature. Hence, we measured the stomatal pore sizes in control and *CaDTR1*-silenced plants after treatment with 20 μM ABA. Consistent with the leaf temperature, the stomatal apertures of *CaDTR1*-silenced plants were larger than those of control plants (Fig. 4f,g).

### Enhanced ABA sensitivity of *CaDTR1*-overexpressing Arabidopsis plants

**Figure 5:**
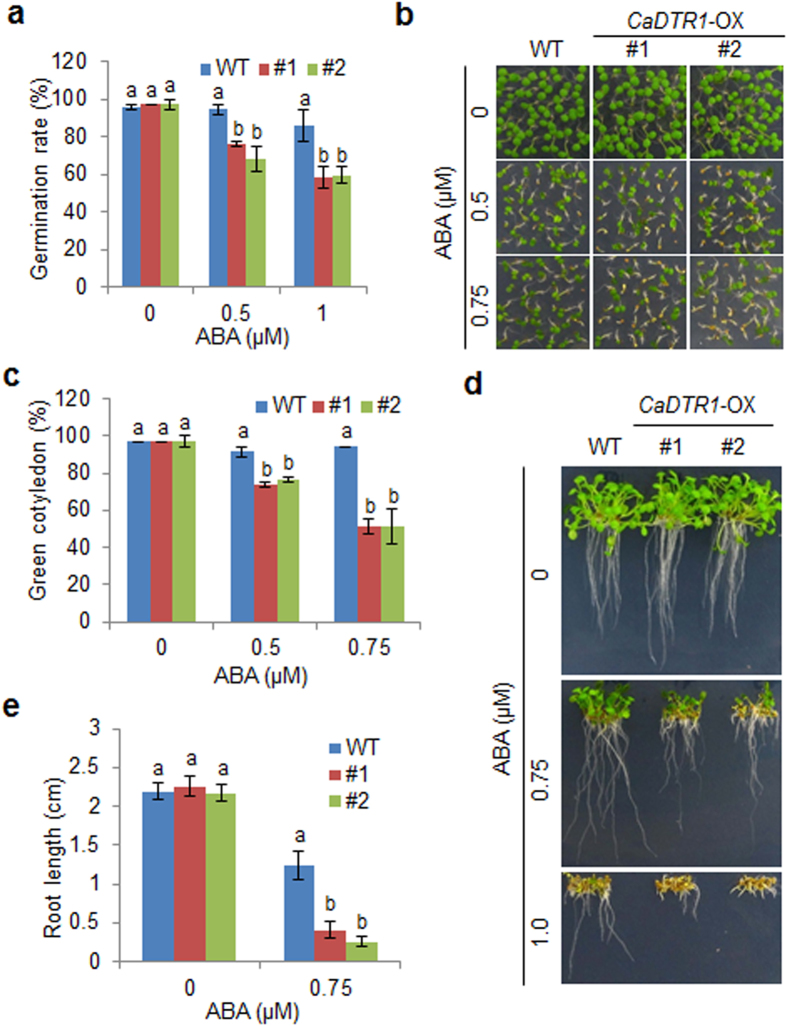
Increased sensitivity of *CaDTR1*-overexpressing (OX) transgenic Arabidopsis plants to abscisic acid (ABA) during germination and seedling growth. (**a**) Seed germination of wild-type (WT) and transgenic lines in response to ABA. Seeds were germinated on 0.5× MS agar plates containing 0.5 μM or 1.0 μM ABA. (**b**,**c**) Growth of WT and transgenic seedlings on 0.5× MS agar plates containing various concentrations of ABA. Representative photographs were taken 5 days after plating. Quantification of green cotyledons in the wild-type and each mutant line was performed 5 days after plating. Data represent the mean ± standard error values obtained after evaluating 72 seeds from three independent experiments. (**d**,**e**) Root elongation of WT and transgenic plants in response to ABA. The root length of each plant was measured 8 days after plating. Data represent the mean ± standard error of three independent experiments. Different letters indicate significant differences in three independent experiments (ANOVA; *P* < 0.05).

**Figure 6:**
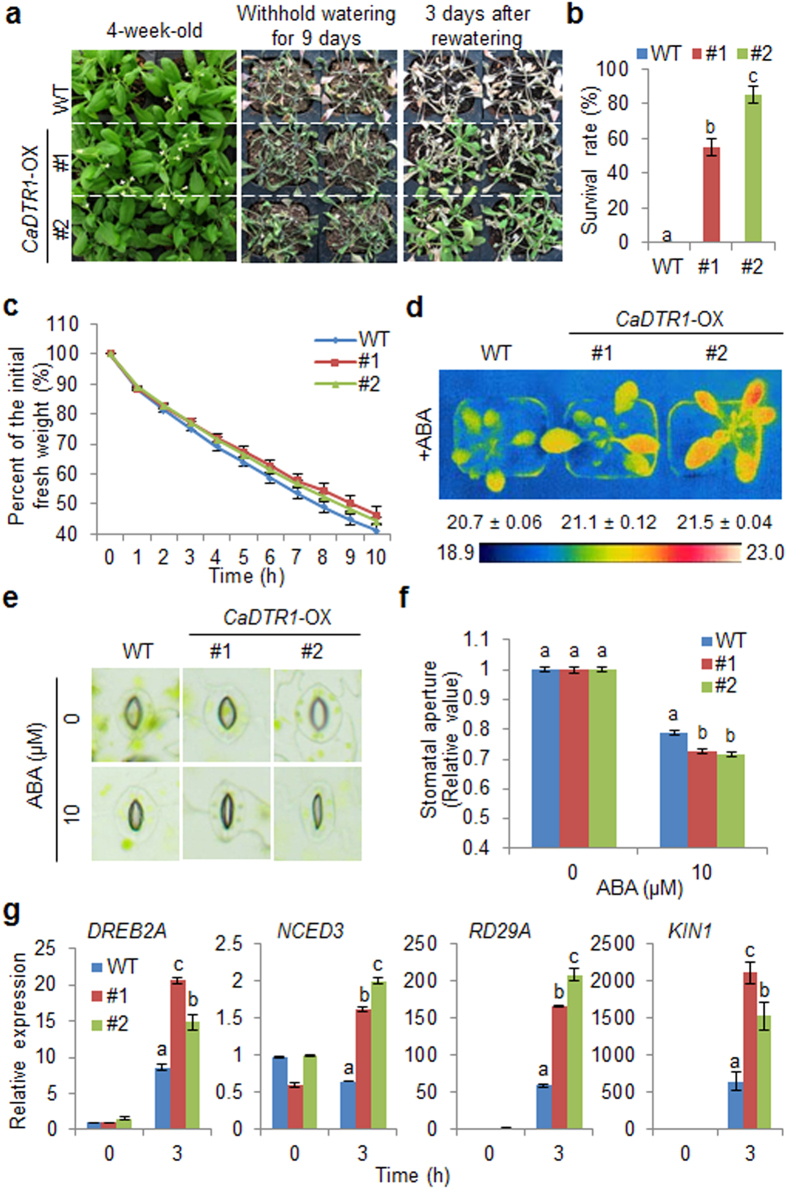
Enhanced tolerance of *CaDTR1*-OX plants to drought stress. (**a**) Drought-tolerant phenotype of *CaDTR1*-OX transgenic plants. Four-week-old WT and transgenic plants were subjected to drought stress by withholding watering for 9 days and then re-watering for 3 days. Representative images were taken before (left) and after (middle) drought and after 3 days of re-watering (right). (**b**) Survival rates of plants after 3 days of re-watering. Data represent the mean ± standard error of three independent experiments, each evaluating 20 plants. (**c**) Transpirational water loss from the leaves of WT and transgenic plants at various times after detachment of leaves. (**d**) Increased leaf temperatures of *CaDTR1*-OX plants in response to ABA treatment. (**e**,**f**) Stomatal apertures in WT and *CaDTR1*-OX plants treated with ABA. Leaf peels were harvested from the 3-week-old plants of each line and incubated in stomatal opening solution (SOS) buffer containing 0 μM or 10 μM ABA. Representative images were taken under a microscope and the stomatal apertures were measured. Data represent the mean ± standard error of three independent experiments. (**g**) qRT-PCR analysis of drought-inducible genes in the *CaDTR1*-*OX* mutant in response to drought stress at 3 h after detachment. The relative expression (ΔΔCT) of each gene was normalized to that of *Actin 8*, used as an internal control gene. Data represent the mean ± standard deviation values from three independent experiments. Different letters indicate significant differences in three independent experiments (ANOVA; *P* < 0.05).

*CaDTR1*-silenced pepper plants exhibited a drought-sensitive phenotype. Hence, we performed additional expression experiments to investigate the potential relationship between *CaDTR1* expression and abiotic stress tolerance. To provide evidence for the role of CaDTR1 in response to abiotic stress, we created transgenic Arabidopsis plants overexpressing the *CaDTR1* gene under the control of the cauliflower mosaic virus (CaMV) 35S promoter. The results of semi-quantitative PCR analysis revealed that the *CaDTR1* gene was not expressed in wild-type plants, but was strongly expressed in two independent T_3_ homozygous transgenic plants (Supplementary Fig. S2). We used these transgenic plants for our phenotypic analyses of plant response to abiotic stresses. Under favourable conditions, we observed no phenotypic differences between transgenic and wild-type plants (Figs 5 and 6).

Seed germination is regulated by plant hormones, including gibberellins and ABA; moreover, the response to abiotic stress is controlled by ABA. Hence, we investigated phenotypic differences between wild-type and transgenic plants in response to ABA (Fig. 5). First, we germinated CaDTR1-OX seeds MS agar medium supplemented with various concentrations of ABA. In the presence of ABA, the germination rate of CaDTR1-OX seeds was significantly lower than that of wild-type seeds (Fig. 5a). Next, we analysed seedling establishment of wild-type and CaDTR1-OX plants in response to ABA (Fig. 5b,c). In the absence of ABA, the rate of cotyledon greening did not differ significantly between wild-type and transgenic plants. However, consistent with the germination rate, in the presence of ABA, the rate of cotyledon greening was significantly lower in CaDTR1-OX plants than in wild-type plants. Finally, we assessed primary root growth in response to ABA (Fig. 5d,e). We found that in the presence of ABA, primary root growth was inhibited in a concentration-dependent manner. Moreover, primary root growth was more strongly inhibited in CaDTR1-OX plants than in wild-type plants. Our results indicate that conferred expression of CaDTR1 in Arabidopsis plants leads to enhanced ABA sensitivity during the germinative and seedling growth stages.

### Enhanced drought tolerance of *CaDTR1*-overexpressing Arabidopsis plants

*CaDTR1*-silenced pepper plants displayed a drought-sensitive phenotype (Fig. 4) and *CaDTR1*-OX plants exhibited an ABA-hypersensitive phenotype (Fig. 5). Hence, we investigated whether *CaDTR1*-OX plants exhibited an altered phenotype in response to drought stress (Fig. 6). We subjected *CaDTR1*-OX plants to drought stress by withholding watering for 9 days and then re-watering for 3 days. Under well-watered conditions, we observed no phenotypic differences between transgenic and wild-type plants (Fig. 6a, left panel). However, after drought stress treatment, wild-type plants displayed a more wilted phenotype than transgenic plants (Fig. 6a, middle panel). In addition, after re-watering, *CaDTR1*-OX plants recovered more rapidly than wild-type plants (Fig. 6a, right panel). At 3 days after re-watering, the survival rate of *CaDTR1*-OX plants was 55–85%, whereas that of wild-type plants was approximately 0% (Fig. 6b). To investigate whether the drought-tolerant phenotype displayed by *CaDTR1*-OX plants is associated with altered water retention, we measured the fresh weight of detached rosette leaves as an indirect indication of the transpiration rate (Fig. 6c). We found that the transpirational water loss was lower in *CaDTR1*-OX plants than in wild-type plants, indicating that the drought-tolerant phenotype is derived from enhanced capacity for water retention.

Generally, drought sensitivity and drought tolerance are assessed using at least two cellular or molecular parameters. Several studies have determined ABA sensitivity, which leads to enhanced drought tolerance, by measuring the leaf temperature and stomatal pore size^24^,^37^,^38^. In addition, the defence response to drought stress is associated with altered expression levels of stress-related genes^24^,^39^. Hence, we measured the leaf temperature, which decreases when the stomata are open, because of evaporative cooling (Fig. 6d). In the presence of ABA, the leaf temperatures were significantly higher in *CaDTR1*-OX plants than in wild-type plants, indicating that conferred expression of *CaDTR1* increases ABA sensitivity, thereby leading to stomatal closure. In the absence of ABA, we determined no significant difference in stomatal pore size between wild-type and transgenic plants; however, in the presence of ABA, the degree of stomatal closure was greater in *CaDTR1*-OX plants than in wild-type plants (Fig. 6e,f). Our results indicate that *CaDTR1*-OX plants exhibit an ABA-hypersensitive phenotype, and this presumably leads to increased water retention under water-deficit conditions.

The expression of stress-responsive genes is related with stress tolerance; hence, we performed qRT-PCR analysis with wild-type and *CaDTR1*-OX leaves treated with drought stress by detachment (Fig. 6g). After 3 h of drought stress, the expression levels of stress-responsive genes, including *DREB2A*, *NCED3*, *RD29A* and *KIN1*, significantly higher in *CaDTR1*-OX leaves than in wild-type leaves.

## Discussion

In the present study, we isolated an ABA- and abiotic stress-inducible gene, *CaDTR1*, which encodes a RING type E3 ligase. The hydrophobic regions and RING finger motif of CaDTR1 contribute to subcellular localisation and ubiquitin ligase activity, respectively. We used gain-of-function genetic studies with *CaDTR1*-OX Arabidopsis plants to show that CaDTR1 positively regulates the stomatal aperture and transpirational water loss, thereby leading to enhanced drought tolerance. We further demonstrated that these *CaDTR1*-OX Arabidopsis plants expressed high levels of ABA-synthesis related genes, stress-responsive genes, and transcription factors in response to drought stress. In addition, we conducted loss-of-function genetic studies with *CaDTR1*-silenced pepper plants and found that these plants displayed an ABA-insensitive and drought-sensitive phenotype. Taken together, our findings indicate that CaDTR1 is a RING-type E3 ligase that confers ABA sensitivity and drought tolerance in plants.

An increasing number of studies have suggested that the ubiquitin–proteasome system is involved in regulation of the stress-signalling pathway at multiple steps^32^,^40^. Several abiotic stress tolerance-related RING type E3 ligases have been isolated and functionally characterised; nevertheless, the precise molecular mechanism whereby RING type E3 ligase activity is regulated remains to be fully elucidated. The RING domain is a common motif found in all eukaryotes, and it plays a key role in the defence response to stress. Moreover, post-translational degradation by RING-type E3 ligases leads to changes in the response to ABA^41^. ABA constitutes an integral part of adaptive responses to drought stress; moreover, when plants encounter water-deficit conditions, ABA levels increase in the plant cells, particularly the guard cells^42^,^43^,^44^. ABA induces stomatal closure by reducing the turgor pressure in the guard cells, thereby regulating transpirational water loss and leading to enhanced drought tolerance^24^,^45^,^46^. Several studies have indicated that RING type E3 ligases function as positive or negative regulators of ABA. Several RING type E3 ligases, such as SDIR1, OsCTR1, XERICO, Rha2a, and Rha2b, function as positive regulators of ABA signalling^27^,^30^,^47^,^48^. In contrast, CaAIR1, RSL1, RGLG2, and AIP2 function as negative regulators of ABA signalling^24^,^28^,^49^,^50^. The results of our sequence analysis and *in vitro* ubiquitination assay imply that CaDTR1 displays E3 ligase activity and may be involved in degradation of target proteins. Our findings indicate that CaDTR1 functions as a positive regulator of ABA signalling and the drought stress response; hence, it is presumably able to degrade target proteins, which act as negative regulators of the drought stress response.

Expression levels of stress marker genes and transcription factors are known to be closely related to stress tolerance^43^,^51^,^52^,^53^,^54^. In the present study, we were unable to identify the substrate target protein; nevertheless, we elucidated changes in expression levels of stress-related genes. In comparison with wild-type plants, *CaDTR1*-OX plants expressed high levels of drought stress marker genes and transcription factors in response to drought stress (Fig. 6g), indicating that CaDTR1 functions in the drought stress response. In particular, we found that the *NCED3* gene was strongly induced by drought stress in *CaDTR1*-OX plants, but determined no significant difference in expression levels between wild-type and *CaDTR1*-OX plants under well-watered conditions. Under water-deficit conditions, *NCED3* functions in ABA biosynthesis and positively regulates transcription of stress-responsive genes, which are crucial for the drought stress response^55^,^56^. Moreover, the expression of *NCED3* is induced by drought stress^56^,^57^. The enhanced expression of *NCED3* shown by *CaDTR1*-OX plants in response to drought stress does not explain whether this gene affects ABA biosynthesis; however, *NCED3* expression may be involved in the expression of other stress-responsive genes and may therefore indirectly contribute to the drought-tolerant phenotype.

In summary, we propose that the RING-type E3 ligase CaDTR1 functions as a positive regulator of the drought stress response in pepper plants via ABA-mediated signalling. *CaDTR1*-silenced pepper plants and *CaDTR1*-OX Arabidopsis plants exhibited ABA-insensitive and ABA-hypersensitive phenotypes, respectively, and these phenotypes displayed altered responses to drought stress. Our findings provide a valuable insight into the plant defence mechanism that operates during drought stress signalling and will therefore facilitate the selection of plants adapted to drought stress. Further studies to identify the target proteins controlled by the CaDTR1–26S proteasome system, and thus to clarify the fine-tune regulation of drought stress via the ABA-dependent signalling pathway, are required.

## Methods

### Plant material and growth conditions

Seeds of pepper (*Capsicum annuum* L., cv. Nockwang) and tobacco (*Nicotiana benthamiana*) were sown in a steam-sterilised compost soil mix (peat moss, perlite, and vermiculite, 5:3:2, v/v/v), sand, and loam soil (1:1:1, v/v/v). The pepper plants were raised in a growth room at 27 ± 1 °C under white fluorescent light (80 μmol photons·m^−2^·s^−1^; 16 h per day). The tobacco plants were maintained in a growth chamber at 25 ± 1 °C under a 16-h light/8-h dark cycle. *Arabidopsis thaliana* (ecotype Col-0) plants were germinated on Murashige and Skoog^58^ (MS) salt supplemented with 1% sucrose (Duchefa Biochemie); the plates were incubated in a growth chamber at 24 °C and under a 16-h light/8-h dark cycle. The *Arabidopsis thaliana* seedlings were maintained in a steam-sterilised compost soil mix (peat moss, perlite, and vermiculite, 9:1:1, v/v/v) under controlled environmental conditions as follows: 24 °C and 60% relative humidity under fluorescent light (130 μmol photons·m^−2^·s^−1^) with a 16-h light/8-h dark cycle. All seeds were vernalised at 4 °C for 2 days before being placed in the growth chamber.

### Sequence analysis

The deduced amino acid sequences for CaDTR1 and its homologs were identified using BLAST searches (http://www.ncbi.nlm.nih.gov/BLAST). SMART (http://smart.embl-heidelberg.de/) and TMHMM (http://www.cbs.dtu.dk/services/TMHMM/) web servers were used to identify the RING finger motif and hydrophobic regions, respectively. In addition, a hydrophobicity plot was constructed using ProtScale (http://web.expasy.org/protscale). The amino acid alignment was performed using ClustalW2 (http://www.ebi.ac.uk/Tools/msa/clustalw2), and the results were edited using Genedoc software (http://www.nrbsc.org/gfx/genedoc). The amino acid alignments were manually regulated to compare the cDNA clones of *CaDTR1* with those of other organisms.

### Generation of *CaDTR1*-overexpressing Arabidopsis plants

The full-length cDNA sequence of *CaDTR1* was integrated into the pK2GW7 binary vector, to induce constitutive expression of the *CaDTR1* gene under the control of the cauliflower mosaic virus (CaMV) 35S promoter in Arabidopsis. The *CaDTR1* binary vectors were transformed into *Agrobacterium tumefaciens* strain GV3101. *Agrobacterium*-mediated transformation of *Arabidopsis thaliana* with the *CaDTR1* gene was performed using the floral dip method^59^. For selection of *CaDTR1*-OX lines, seeds harvested from the putative transformed plants were sown on MS agar plates containing 50 μg·mL^−1^ kanamycin.

### Subcellular localisation analysis

The coding regions of the *CaDTR1* gene without stop codon were inserted into the binary vector p326GFP. *Agrobacterium tumefaciens* strain GV3101 carrying this construct was combined with the p19 strain (1:1 ratio; OD_600_ = 0.5) and co-infiltrated into fully expanded leaves of 5-week-old *N. benthamiana*. At 2 days after infiltration, microscopic analysis was performed.

### Virus-induced gene silencing

The tobacco rattle virus (TRV)-based virus-induced gene silencing (VIGS) system was used to generate *CaDTR1* knockdown in pepper plants^24^. A 420–630-bp fragment of the *CaDTR1* cDNA was inserted into the pTRV2 vector to generate pTRV:*CaDTR1*. *Agrobacterium tumefaciens* strain GV3101 containing pTRV1, pTRV2:00, and pTRV:*CaDTR1* was co-infiltrated into the fully expanded cotyledons of pepper plants (OD_600_ = 0.2 for each construct).

### Phenotypic analyses of responses to ABA and drought treatments

For germination tests, 100 seeds per genotype were sown on plates containing MS agar medium supplemented with various concentrations of ABA. The number of seeds showing radicle emergence were counted. For root growth assays during the post-germinative stage, germinated seeds were transferred to plates containing MS agar medium supplemented with various concentrations of ABA. The root lengths of the seedlings were measured. Drought tolerance assays were conducted as described by Lim and Lee^60^. One-week-old seedlings of wild-type and *CaDTR1*-OX lines were randomly planted in pots containing soil mixture (9:1:1 ratio of peat moss, perlite, and vermiculite) and grown under normal watering conditions for 2 weeks. To impose drought stress, watering was withheld for 9 days. The plants were then re-watered for 3 days and the survival rates were calculated. For measuring transpirational water loss from the leaves, 10 leaves were detached from 3-week-old plants of each line and placed in Petri dishes. The dishes were placed in a growth chamber at 40% relative humidity, and the loss of fresh weight was determined at the indicated time points. Each experiment was performed in triplicate.

### Stomatal aperture bioassay

The stomatal aperture bioassay was conducted as described previously^61^, but with some modifications. Leaf peels were collected from the rosette leaves of 3-week-old plants and were floated in a stomatal opening solution (SOS; 50 mM KCl, 10 mM MES-KOH, 10 μM CaCl_2_, pH 6.15). The peels were incubated for 3 h to obtain >80% stomatal opening in Arabidopsis Col-0 plants. The buffer was replaced with fresh SOS containing various concentrations of ABA. Leaf peels were then incubated for a further 3 h. In each individual sample, 100 stomata were randomly observed under a Nikon Eclipse 80i microscope. The widths and lengths of individual stomatal pores were recorded using Image J 1.46r software (http://imagej.nih.gov/ij). Each experiment was performed in triplicate.

### RNA isolation and semi-quantitative reverse transcription-polymerase chain reaction

Total RNA was isolated from the leaf tissues of *Arabidopsis thaliana* plants using an RNeasy Mini kit (Qiagen, Valencia, CA, USA). To remove genomic DNA, all RNA samples were digested with RNA-free DNase. After quantification using a spectrophotometer, 1 μg of total RNA was used to synthesise cDNA using a Transcript First Strand cDNA Synthesis kit (Roche, Indianapolis, IN, USA) according to the manufacturer’s instructions. Concomitantly, cDNAs were synthesised without reverse transcriptase and were subjected to semi-quantitative RT-PCR to eliminate the possibility of contamination by genomic DNA in the cDNA samples. Semi-quantitative RT-PCR analysis was performed using Ex-taq DNA polymerase and specific primers. Each reaction was performed in triplicate. The PCR was programmed as follows: 95 °C for 5 min; 45 cycles at 95 °C for 20 s; 58 °C for 20 s; and 72 °C for 20 s. Arabidopsis *Actin 8* was used as an internal control^62^.

### *In vitro* ubiquitination

The procedure for the expression and purification of the maltose-binding protein (MBP)–CaDTR1 recombinant protein is described in Park *et al*.^24^. For the *in vitro* ubiquitination assay, the purified MBP–CaDTR1 (500 ng) was mixed with ubiquitination reaction buffer [50 mM Tris-HCl, pH 7.5, 10 mM MgCl_2_, 0.05 mM ZnCl_2_, 1 mM Mg-ATP, 0.2 mM DTT, 10 mM phosphocreatine, and 0.1 unit of creatine kinase (Sigma-Aldrich)] containing 250 ng of recombinant human UBE1 (Boston Biochemicals, Cambridge, MA, USA), 250 ng of recombinant human H5b (Enzo Life Sciences, Farmingdale, NY), and 10 μg of bovine ubiquitin (Sigma-Aldrich). After incubation at 30 °C for 3 h, the reacted proteins were separated using SDS-PAGE and analysed using immunoblotting with anti-ubiquitin antibody (Santa Cruz Biotechnology, Santa Cruz, CA) and anti-MBP antibody (New England Biolabs, Ipswich, MA).

## Additional Information

**How to cite this article**: Joo, H. *et al*. Identification and functional expression of the pepper RING type E3 ligase, CaDTR1, involved in drought stress tolerance via ABA-mediated signalling. *Sci. Rep.* **6**, 30097; doi: 10.1038/srep30097 (2016).

## Supplementary Material

Supplementary Information
