# Supplementary material for: Identification and functional expression of the pepper RING type E3 ligase, CaDTR1, involved in drought stress tolerance via ABA-mediated signalling
Source: Sci Rep. 2016 Jul 21;6:30097. doi: 10.1038/srep30097 (PMC4954983; doi:10.1038/srep30097)
Supplement: Supplementary Information [file srep30097-s1.pdf]

Supplementary data

Identification and functional expression of the pepper RING type E3 ligase, CaDTR1, involved in drought stress tolerance via ABA-mediated signalling

Hyunhee Joo, Chae Woo Lim, Sung Chul Lee

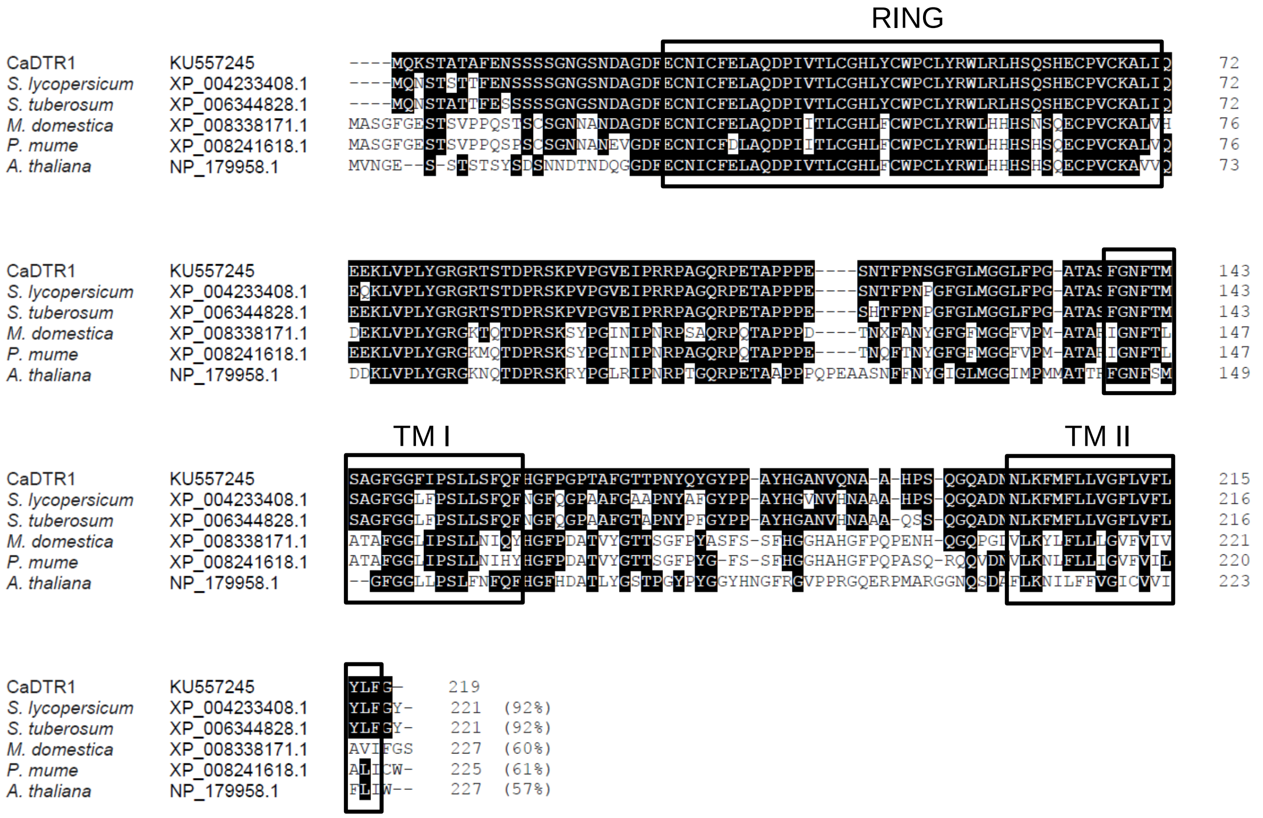

Supplementary Figure S1. Comparisons of the deduced amino acid sequence of the CaDTR1 protein (accession no. KU557245) with those of *Solanum lycopersicum* (accession no. XP_004233408.1), *Solanum tuberosum* (accession no. XP_006344828.1), *Malus domestica* (accession no. XP_008338171.1), *Prunus mume* (accession no. XP_008241618.1), and *Arabidopsis thaliana* (accession no. NP_179958.1) proteins. Identical amino acid residues are highlighted in black; Boxes reveal the Really Interesting New Gene (RING) zinc finger domain and transmembrane domains.

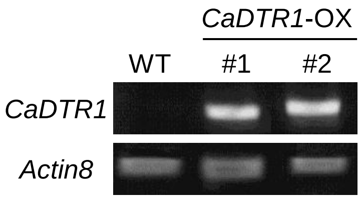

Supplementary Figure S2. RT-PCR analysis of *CaDTR1* expression in wild-type and *CaDTR1*-OX transgenic lines. *Actin8* was used as an internal control gene.
